# Surgical Expertise in Neonatal Extracorporeal Membrane Oxygenation (ECMO): A Single Center Experience

**DOI:** 10.3389/fped.2019.00398

**Published:** 2019-09-27

**Authors:** Francesco Macchini, Antonio Di Cesare, Anna Morandi, Martina Ichino, Genny Raffaeli, Federica Conigliaro, Gabriele Sorrentino, Simona Neri, Fabio Mosca, Ernesto Leva, Giacomo Cavallaro

**Affiliations:** ^1^Department of Pediatric Surgery, Fondazione IRCCS Ca' Granda Ospedale Maggiore Policlinico, Milan, Italy; ^2^Neonatal Intensive Care Unit, Fondazione IRCCS Ca' Granda Ospedale Maggiore Policlinico, Milan, Italy; ^3^Department of Clinical Sciences and Community Health, Università degli Studi di Milano, Milan, Italy; ^4^Betamed Perfusion Service, Rome, Italy; ^5^Pediatric Anesthesiology and Intensive Care Unit, Department of Anesthesia and Critical Care, Fondazione IRCCS Ca' Granda Ospedale Maggiore Policlinico, Milan, Italy

**Keywords:** neonatal, child, ECMO, surgery, training

## Abstract

**Introduction:** The surgical technique for peripheral cannulation aimed at providing extracorporeal membrane oxygenation (ECMO) is well described. Training methods for surgeons still need proper standardization, especially in newborn patients. This study aims to evaluate the surgical training outcomes of a neonatal ECMO team.

**Materials and Methods:** A 4 year training program (2014–2018) was developed to achieve the skills in the surgical technique for neonatal veno-arterial ECMO. Surgeons with experience in neonatal and vascular surgery were selected for the training. The training consisted of educational sessions, high-fidelity simulations, *in vivo* swine model procedures, international fellowship, and periodical simulations. The preliminary clinical experience in surgical neonatal ECMO management (2016-present) was analyzed by recording the following data: indications for ECMO and patients' data; effectiveness of cannulations (number; perioperative complications of cannulation; major surgical events during ECMO); efficacy of decannulation (number and perioperative complications).

**Results:** 12 neonates (5 females) fitted the ELSO criteria for ECMO. Nine newborns were affected by CDH; 1 by H1N1 flu-related pneumonia; 1 by meconium aspiration syndrome and one by Respiratory Syncytial Virus related bronchiolitis. Mean weight at cannulation was 3,281 g (range 2,330–3,840 g); mean gestational age was 36 weeks. No procedure was aborted, and no intra-operatory mortality was recorded. Mean operative time was 86 ± 30 min. The caliber of the carotideal cannulas ranged from 8F (8 patients) to 10F (2 patients); the caliber of the jugular cannulas were: 8F cannula (2 patients), 10F (6 patients), and 12F (2 patients). Four complications occurred: a case of air in the circuit, two cases of azygous vein cannulation and a partial dislocation of the venous cannula during the daily care maneuvers. All of them were promptly recognized and successfully treated. The mean ECMO duration was 7.1 ± 4.2 days (range 2–16 days). Seven patients (78%) were decannulated effectively. Mean decannulation time was 53 min (range 45–80 min). No complications occurred during the decannulation process. No ECMO–related deaths were recorded.

**Conclusions:** Neonatal respiratory ECMO still represents a challenge. Experienced neonatal surgeons can manage the neck vascular cannulation. The codified procedure must be adhered to after appropriate training and following a proper learning curve.

## Introduction

Extracorporeal membrane oxygenation (ECMO) is an advanced life support therapy for life-threatening pulmonary and cardiac diseases in children (1). ECMO is a high-risk procedure and requires quick and effective decisions based on well-established knowledge as well as technical and organizational proficiency in an emergency setting (2). Despite the advances in equipment and technology, the adoption of standardized protocols and preventive strategies, and the increasing experience of providers, neonatal ECMO still remains a challenging procedure, due to its intrinsic high morbidity and mortality (3).

Accordingly, the Extracorporeal Life Support Organization (ELSO) strongly supports specific programs for education and training in order to improve patients' outcomes (4). Multidisciplinary team training has been previously described in the neonatal and pediatric ECMO setting (5, 6). Simulation-based cannulation curricula have been reported in the cardiac, pediatric setting (7).

Although the surgical technique for peripheral VA-ECMO cannulation is well-described (8), surgical training methods to acquire and master the procedure still need standardization.

Regarding neonatal respiratory ECMO, the most common indications are congenital diaphragmatic hernia (CDH), meconium aspiration syndrome (MAS), pulmonary hypertension (PPHN), sepsis, and respiratory distress syndrome (RDS). The veno-arterial (VA) cannulation is the selected method in most Neonatal Intensive Care Units (NICU) (8).

ECMO-related mortality is still high in neonates, with only a 73% survival to hospital discharge (SHD) according to the ELSO registry reports (9) and a 57% according to a single center experience (3).

Mortality is particularly high for CDH, which have an SHD rate of 50%, as compared to MAS (92%) and PPHN (73%) (9). An early start of ECMO within 24 h after birth and a reduced time of ECMO duration (<7 days) seems effective in leading to SHD independently of other factors (3).

Next, with regards to survival rates, efforts are needed to be shifted toward the improvement of ECMO patients' outcomes (10).

The overall complication rate of ECMO in newborns is 49% (9). The ELSO registry reports 54.5% of surgical complications, with hemorrhagic ones accounting for 26.2%, mechanical ones for 16.2%, pulmonary ones for 10.6% and cardiovascular ones for 1.5% (9).

The aim of this study is to evaluate the clinical impact of surgical team training, in terms of technical performance of VA peripheral cannulation and decannulation in newborns treated by a single team of neonatal surgeons since the establishment of a new ECMO program.

## Materials and Methods

We conducted a retrospective, single-center, cohort study at the NICU of the Department of Clinical Sciences and Community Health, Fondazione IRCCS Ca' Granda Ospedale Maggiore Policlinico in Milan, Italy. No modifications were made to the standard of care for newborns requiring respiratory ECMO. Ethics committee approval was not required for this observational retrospective study on a small series of patients. Parents gave written informed consent for the publication of this series in accordance with the Declaration of Helsinki.

### Training Curriculum

The ELSO guidelines, the “ELSO Redbook” and the “ELSO ECMO Specialist Training Manual” were selected as reference guidelines for our Institute (4, 8, 11).

The VA cannulation was selected as the preferred modality in our Institute for neonatal respiratory ECMO.

A 4-year training program (September 2014–2018) was delivered to achieve surgical skills in neonatal VA ECMO. The following multistep training program was designed for the whole ECMO team to reach full autonomy. Only surgeons with at least 2 years of experience and more than 100 procedures of neonatal and vascular surgery were selected for the training after being evaluated by the head of Department. The surgical training was supported by the implementation of a larger multi-disciplinary training program (5). As previously reported, the training process started with the delivery of educational material, attendance to national and international courses, and observership programs at ECMO referral centers. Next, with regards to the educational session, hands-on training was delivered through “wet labs,” animal-based, and mannequin-based high-fidelity simulation sessions.

During the whole training process, attention was focused on both technical and behavioral factors that would impact the patient's outcome. Each session ended with extensive debriefing and immediate feedback.

The “wet labs” aimed to familiarize the participants with equipment and procedures. They consisted of hands-on training on a closed-loop ECMO circuit filled with saline. The animal laboratory consisted of isolating and cannulating neck vessels on anesthetized piglets to start a VA ECMO. During the ECMO run, all members of the team, including pediatric surgeons, were involved in routine practices. According to the participants, this step appeared more similar to reality and crucial from a surgical point of view to develop adequate technical skills.

High-fidelity simulation sessions were performed with a neonatal mannequin (SimNewB® Laerdal). Tubes were inserted in the right side of the neck and distally connected to a reservoir bag, filled with fake blood.

Once the surgeon in training had completed the whole program, having effectively cannulated at least 10 piglets, they were considered for the clinical surgical approach (5). Simulations were carried on systematically even after treating the first patients in order to maintain a high level of technical ability.

### Surgical Procedure

VA-ECMO was offered, based on ELSO criteria (11). Based on our institutional protocol, we considered neonates weighing over 2 kg and with a post-gestational age of 34 weeks (12) as eligible for ECMO.

Vascular access was obtained by a right neck incision and cannulation of the internal jugular vein and carotid artery. Cannulation was always performed in a dedicated and isolated room of the NICU. Adequate exposure of the neck was obtained by putting a small roll transversely beneath the shoulders of the neonate and by a minor contralateral rotation of the head. Deep sedation/anesthesia with muscle relaxation was required to prevent spontaneous breathing. After a transverse cervical incision, a complete exposition of the vessels was obtained. Titration of heparin occurred before placing the cannula every 3 min following boluses of 25 IU/kg, to achieve an activated clotting time of 220–250 s. The average dose was 50 IU/kg, while the maximum cumulative dose was 100 IU/Kg.

**Figure 1:**
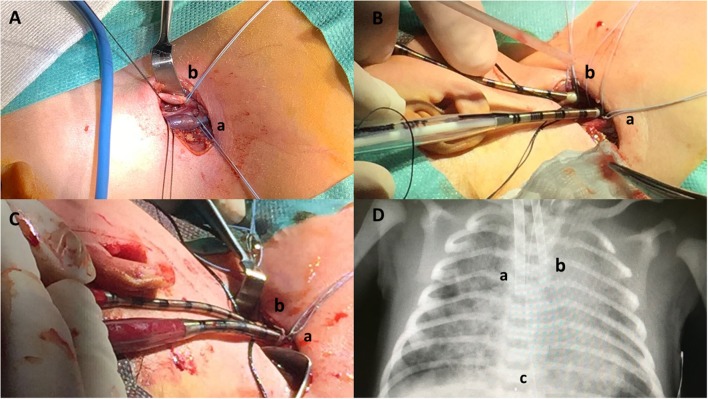
Neonatal veno-arterial Surgical cannulation site in the neck for respiratory ECMO: surgical steps. **(A)** Neck skin incision and isolation of internal jugular vein **(a)** and carotid artery **(b)**. **(B)** Vascular cannulation. Drainage cannula (10 French) inserted in the jugular vein **(a)**, inflow cannula (8 French) inserted in the carotid artery **(b)**. **(C)** ECMO start. Drainage cannula (10 French) inserted in the jugular vein **(a)**, inflow cannula (8 French) inserted in the carotid artery **(b)**. **(D)** Chest X-ray. Drainage cannula (10 French) inserted in the jugular vein **(a)**, inflow cannula (8 French) inserted in the carotid artery **(b)**, and cannula tip **(c)**.

Cannula selection occurred after cut down with direct inspection of the vessels; the biggest caliber of both arterial and venous cannulas was chosen according to the patient's vessel sizes, maximal blood flow needed and the pressure drop of the cannula itself. Therefore, the Single Lumen Venous (8F, 10F, 12F, or 14F) and Arterial (8F, 10F, or 12F) cannulas (Bio-Medicus®, Medtronic), heparin-based coated (Carmeda® BioActive Surface), were selected for our neonates. Non-absorbable braided 4.0 ligatures were slid around the vessels above and below the cannulation site (Figure 1A). The cephalic ends of the vessels were ligated. The vessels were opened and the cannulas placed. The venous cannula was inserted for 6–9 cm while the arterial one for 3–5 cm, according to the size of the newborn (Figure 1B). Cannulas were then connected to the ECMO circuit, carefully avoiding air embolism (Figure 1C). Chest X-ray was performed to verify the position of the tip of the cannula after ECMO support was initiated, and minor cannula position adjustments were made (Figure 1D). Intraoperative echocardiography with Doppler flow was implemented to assess the position of the tip of the cannula in the right atrium. Once the correct position was confirmed, the cannulas were secured to the muscle insertions and to the retro-auricular skin to prevent dislocation.

At the time of decannulation, ligation of the neck vessels was the chosen option in our Center (8).

### Surgical Outcome

To evaluate the impact of the surgical expertise on ECMO cannulation, we retrospectively analyzed our case-series from the establishment of the ECMO program (March 2016) to present (March 2019). We retrieved the following data: indications to ECMO and demographic data; effectiveness of cannulation; caliber of venous and arterial cannulas; perioperative complications of cannulation; major surgical events during ECMO; effectiveness of decannulation and perioperative complications of decannulation.

As reported by the ELSO Registry, complications are classified as (1) mechanical (oxygenator failure, raceway rupture, other tubing rupture, pump failure, circuit air, cannula-related problems); (2) hemorrhagic (cannulation site bleeding, surgical site bleeding); (3) cardiovascular (CPR required, cardiac arrhythmia, tamponade); (4) pulmonary (pneumothorax, pulmonary hemorrhage); (5) infectious (9). We considered only the surgical complications.

The severity of every complication was assessed according to the Clavien-Dindo Classification of Surgical Complications (Table 1). It is a morbidity scale based on the therapeutic consequences of complications, that constitutes a simple, objective, and reproducible approach for comprehensive surgical outcome assessment (13).

**Table 1:** Classification of surgical complications grades definition (modified from Clavien-Dindo Classification).

Grade I	Any deviation from the normal post-operative course without the need for pharmacological treatment or surgical, endoscopic, and radiological interventions. Acceptable therapeutic regimens are: drugs as antiemetics, antipyretics, analgesics, diuretics and electrolytes and physiotherapy. This grade also includes wound infections opened at the bedside
Grade II	Requiring pharmacological treatment with drugs other than such allowed for grade I complications. Blood transfusions and total parenteral nutrition are also included
Grade III	Requiring surgical, endoscopic or radiological intervention Grade III-a: intervention not under general anesthesia
Grade III-b	Intervention under general anesthesia
Grade IV	Life-threatening complication (including CNS complications)^‡^ requiring IC/ICU-management
Grade IV-a	Single organ dysfunction (including dialysis)
Grade IV-b	Multi-organ dysfunction
Grade V	Death of a patient

‡ *Brain hemorrhage, ischemic stroke, subarachnoidal bleeding, but excluding transient ischemic attacks (TIA); CNS, central nervous system; IC, intermediate care; ICU, intensive care unit*.

## Results

Three neonatal surgeons completed the training process after 3 years and were considered ready to start with the first patient. In the following year, they continued with the simulation sessions while other surgeons were selected to start the training program. Within the study period, 12 neonates (5 females) fitted the ELSO criteria for ECMO. Nine newborns were affected by CDH; 1 by H1N1 flu-related pneumonia; 1 by meconium aspiration syndrome and one by Respiratory Syncytial Virus (RSV) related bronchiolitis.

The main characteristics of the population are summarized in Table 2.

**Table 2:** Population characteristics.

CDH	- Number of patients - Sex - GA (weeks) - Birth weight (gr) - Site - Defect size - O/E LHR (%) - Diaphragmatic patch (n pts) - Liver up (n pts) - Stomach up (n pts) - Age at ECMO (days) - Weight at ECMO (gr) - ECMO duration (days) - Age at CDH repair (days) - Repair on ECMO (n pts)	9 Males: 5 Females: 4 36.5 ± 1.8 2.660 ± 383 right: 3 left: 6 B: 2 C: 5 D: 2 27 ± 17 7 9 6 8.5 (range 1–52) 3.272 ± 632 6.6 ± 4.1 2.9 ± 1.9 3
H1N1 flu pneumonia	- Number of patients - Sex - GA (weeks) - Birth weight (gr) - Age at ECMO (days) - Weight at ECMO (gr) - ECMO duration (days)	1 Female 32 1.955 53 gr 3.310 12
Meconium aspiration syndrome	- Number of patients - Sex - GA (weeks) - Birth weight (gr) - Age at ECMO (days) - Weight at ECMO (gr) - ECMO duration (days)	1 Male 39 3.635 3 3.840 2
Respiratory Syncytial Virus bronchiolitis	- Number of patients - Sex - GA (weeks) - Birth weight (gr) - Age at ECMO (days) - Weight at ECMO (gr) - ECMO duration (days)	1 Male 34 2.400 30 2.850 6

*Data are expressed as mean ± SD*.

Mean weight at cannulation was 3,281 g (range 2,330–3,840 g); mean gestational age was 36 weeks. No procedure was aborted, and no intra-operatory mortality was recorded. Mean operative time was 86 ± 30 min (from draping to the skin closure including the external fixation of the cannulas and the radiological check). The caliber of the carotideal cannula ranged from 8F (8 patients) to 10F (2 patients); the caliber of the jugular cannula showed a wider range: 8F (2 patients), 10F (6 patients), and 12F (2 patients).

As for the complications, two grade I and two grade III complications occurred (Table 3). Among Grade I complications, a 30-day old male affected by RSV infection presented microbubbles of air in the circuit immediately after connection. The amount of air resulted minimal so that it was promptly fixed through the circuit, without any clinical consequence. The second one, a 1 day old male (GA 39 w; BW 3,280 g) with a right CDH, was cannulated with 8F arterial and 10F venous cannulas. The ECMO support was initiated, but the maintenance of adequate blood flow was impaired by worsening drainage pressure. Despite the administration of multiple fluid boluses, we couldn't reach a satisfactory blood flow support. Echocardiography demonstrated the correct position of the arterial cannula, but the venous cannula was not visualized in the superior vena cava or the right atrium, thus raising the suspicion of azygous vein cannulation. Lateral X-ray of the thorax confirmed the diagnosis of cannula displacement. By modifying the head position, we could reach a 100 ml/kg blood flow, until the decannulation 3 days later.

**Table 3:** Neonatal VA ECMO: classification of complications according to the Clavien-Dindo Classification.

**Grade**	**Cannulation**
I	2 Malposition Microbubble air in circuit
II	–
III	2 Malposition Dislocation
IV	–
V	–
Total (%)	4/12 (33%)

As for grade III complications, a left CDH female (GA 36 w; BW 3,200 g) had a partial dislocation of the 10F venous cannula during the daily care maneuvers. The event was successfully treated, pushing the cannula back inside the jugular vein. ECMO support did not stop. The correct position of the cannula was subsequently confirmed by echocardiography. After this episode an increased level of attention was devoted to the securing of the cannulas.

The last complication occurred in a 1 day old male (GA 37 w; BW 2,530 g) with a right-sided CDH. A 10F venous cannula and an 8F arterial cannula were positioned without any difficulties. As in the previous patient with right CDH, we experienced high drainage pressure, which was not justified by the cannulation caliber and the clinical state of the neonate. Despite fluid therapy, repositioning of the head and multiple attempts to direct the venous cannula into the right atrium, we failed to support the neonate adequately. Consequently, caval cannulation via sternotomy was obtained on day 2 of life and ECMO was maintained for 16 days. The procedure was performed in association with a pediatric cardio-thoracic surgeon to offer the best multi-disciplinary approach.

No other minor or major complications were recorded during the procedure.

The mean ECMO duration was 7.1 ± 4.2 days (range 2–16 days). Seven patients (58%) tolerated the weaning trial of ECMO flow, and they were all decannulated effectively. Mean decannulation time was 53 min (range 45–80 min).

No intra-operative complications occurred during the decannulation process.

Finally, five patients survived to hospital discharge after ECMO treatment (42%). Among the causes of death of the other seven patients, none were ECMO–related. In the CDH population the survival rate was 22%.

## Discussion

ECMO is a well-established advanced life support therapy for critically-ill children with pulmonary or cardiac failure (1). It involves technically challenging procedures and extensive multidisciplinary coordination. Even with skilled providers, ECMO has significant risks of morbidity and mortality (3). Knowledge and routine training are essential to improve patient outcomes, as recommended by ELSO (4, 5).

ECMO clinical specialists (CS) and technicians are the first line of defense in recognizing and responding to acute and potentially life-threatening complications. The ECMO-CS is defined as “the technical specialist trained to manage the ECMO system and the clinical needs of the patient on ECMO”; therefore, their ability to identify and appropriately react to a deteriorating patient is essential for optimal care. The education and training of the ECMO-CS are supported by ELSO, which requires the providers to complete didactics on circuit and patient emergencies (4).

While most centers rely on ECMO-CS to serve as first responders to circuit emergencies, there are no nationally established standards or certifications to provide ECMO care (2). Therefore, the CS, which may include surgeons, nurses, neonatologists, anesthesiologists, and perfusionists, may vary in training and experience depending on local institutional training standards.

As a result, there is wide variation amongst institutions in protocols used to address ECMO circuit emergencies (2).

Due to the different surgical approaches, ECMO for cardiac failure is generally managed by cardiothoracic surgeons with central cannulation, while ECMO for neonatal respiratory diseases is more frequently managed by pediatric surgeons with a neck approach. Respiratory diseases in newborns that may require ECMO include CDH, MAS, and PPHN. These conditions account for almost 75% of all neonatal respiratory ECMO cases. Other diagnostic categories that may constitute indications for ECMO are sepsis (10%) and RDS (5%) (14).

In addition, many Neonatal Departments treating children with pulmonary indications to ECMO are not equipped with pediatric cardiothoracic surgeons, so that different pediatric surgeons' teams have to be involved in the ECMO training and management. In particular, neonatal surgeons were identified as suitable for the neonatal ECMO program in our Center. Only surgeons highly experienced in neonatal and vascular treatment were selected. A 4-year multi-step theoretical and practical training program was required. Only surgeons who completed the training program were considered suitable to manage the cannulation and decannulation procedures in newborns.

As previously described, according to most centers, a VA, as opposed to VV ECMO support, was employed for neonatal respiratory diseases (14).

According to our results, we believe that one of the keys of a successful ECMO cannulation in the neonatal period is based on the strict adherence to standardized surgical procedures. In our opinion, some techniques may help in increasing surgical effectiveness when applying a neck approach. Before surgery, the newborn has to be positioned supine, with neck extension under the shoulders and contralateral head rotation. This position must not be extreme in order to reduce the risk related to cannula progression and malposition at the end of the surgical procedure, when the newborn is placed back in a neutral position. A transverse cervical incision, approximately 3–4 cm in length to achieve a satisfactory exposure of the vessels, is made one finger's breadth above the clavicle over the lower aspect of the right sternocleidomastoid muscle. Direct handling of the vessels has to be minimized to avoid spasm. Finally, while connecting cannulas to the ECMO circuit, a member of the surgical team has to fill both ends of the tubes with saline to prevent air entrapment in the circuit, thus avoiding turbulences and embolism.

As for the decannulation procedure, a high degree of attention is required while managing the arterial cannula, as in newborns it is inserted in the carotid artery only for 3–4 cm and a spontaneous dislocation leading to major bleeding is not unlikely.

Although complications show decreasing trends over time, this therapy remains associated to a high percentage of morbidities and risks (14).

In our study, we decided to adopt a classification of surgical complications in order to make our results comparable to other series. We chose the Clavien-Dindo Classification because, even if it was designed for adults, it has been found to have high sensitivity for pediatric urology, oncology and also neonatal surgery. Its merit is that it moved the focus from the mere occurrence of complications to the grade of complication as a function of the resulting outcome (15).

Even in our preliminary series, complications occurred in 4/12 (33%) patients after cannulation.

In the first ones, two minor complications occurred (Grade I) and were managed without the need for pharmacological or surgical treatment. One of these complications consisted of air entrapment in the circuit, but the amount of air was minimal so that it was immediately fixed through the circuit, without any clinical consequence. Accurate filling of the connections between cannulas and ECMO circuit with saline may prevent such complications.

Clots may also be dangerous both for the circuit, leading to a mechanical obstruction, and the patient, with the risk of embolism. They represent the most common mechanical complication for neonatal respiratory and cardiac ECMO support (9). Clot development in the oxygenator, bridge, and bladder has decreased since 2000, while there has been an increased number in the “Other clots” category. Additional mechanical complications, such as air bubbles in the circuit and oxygenator failures, have been stable to decreasing over time (14). In our series, we did not experience any clot formation during cannulation time.

Also, two complications of medium severity occurred (Grade III), leading to surgery. However, in both cases, problems were effectively solved, with no need for ECMO interruption nor patient instability.

A special mention has to be given to one of the potential complications in the right CDH newborns. As described in a previous paper (16), misplacements of the venous cannulas were also recorded in group 2, with the tip located at the confluence of the azygous vein with the Superior Vena Cava (SVC).

Previous papers suggest that infants with right CDH experience higher morbidity and statistically borderline higher mortality than their left-sided counterparts (17).

Regarding ECMO, the anatomical distortion of the mediastinal vessels that develop as a consequence of right CDH is of particular concern. The combination of a leftward mediastinal shift, intrathoracic liver, and compression of the inferior vena cava (IVC) can perturb the orientation of the great vessels and interfere with venous cannulation. Correct diagnosis of this complication is not always easy, and signs may appear later in the course of ECMO. In our experience, a combination of impaired ECMO flow maintenance with increased drainage pressures, echocardiographic assessment, and cross-table lateral chest X-rays can help to confirm suspected azygous vein cannulation by demonstrating a posteriorly orientated venous cannula (18).

Direct cannulation of the right atrial appendage via sternotomy is an aggressive but feasible method to provide ECMO support in such cases, and may be the only available alternative. In such an occurrence, it may be advisable to seek the collaboration of a cardiothoracic team. Indeed, sternotomy is not frequently needed for respiratory ECMO and the availability of a multidisciplinary team may offer the best results.

The indication must be carefully evaluated according to the effectiveness of ECMO exchanges and the general conditions of the child. As previously suggested by Kuenzler et al., we agree that intraoperative ultrasound guidance can be effective in decreasing the ECMO catheter malposition rate and may prove to be a useful tool, especially in cases where an anatomical variance is suspected (19).

Furthermore, as previously mentioned, limited contralateral head rotation and neck extension may reduce the risks related to cannula progression and malposition at the end of the surgical procedure, when the newborn is placed back in a neutral position.

Our rate of surgical complications seems to be similar to other series (9). However, it is difficult to make a proper comparison because, as far as we know, other series considered surgical and medical complications together. We strongly recommend adopting specific classifications for surgical complications, especially when evaluating the outcome of selected surgeries, such as the neonatal and oncological ones.

In our experience, SHD is lower than the mean value reported in the ECLS Registry Report 2019 (42 vs. 73%), especially considering the CDH population (22 vs. 50%). Nonetheless, no ECMO-related mortalities due to technical issues were recorded in our series. A possible explanation of the low survival rate may be found in the high number of severe CDH newborns in our series, as shown by the low O/E LHR. In fact, a Fetal Endo-Tracheal Occlusion (FETO) program is available in our center and therefore we frequently deal with severe conditions.

In the future, based on a larger population, we should evaluate SHD in different severity groups, according to pre-natal observed/expected lung-to-head ratio (O/E LHR), side and size of the defect.

Conversely, when considering the population other than CDH, the SHD was 100% in our population.

Despite the limited numbers, our results support the value of a standardized training program and a dedicated multidisciplinary team aimed to improve the management of severe cases.

In conclusion, neonatal respiratory ECMO represents a challenge even after prolonged dedicated training. Experienced neonatal surgeons can manage the neck vascular cannulation. The codified procedure must be adhered to after appropriate training and following a proper learning curve. This seems to provide adequate surgical skills, effective execution of ECMO in the neonatal period, and prompt intervention to manage the limited complications, with a positive impact on clinical prognosis.

## Data Availability Statement

The datasets generated for this study are available on request to the corresponding author.

## Ethics Statement

Ethical review and approval was not required for the study on human participants in accordance with the local legislation and institutional requirements. Written informed consent to participate in this study was provided by the participants' legal guardian/next of kin.

## Author Contributions

FMa, GC, GR, AM, MI, EL, and FMo contributed conception and design of the review. FC, AD, SN, and GS collected and analyzed the data retrospectively. FMa, GC, GR, AM, and MI wrote the first draft of the manuscript. All authors contributed to manuscript critical revision, read, and approved the submitted version.

### Conflict of Interest

FC was employed by company Betamed Perfusion Service. The remaining authors declare that the research was conducted in the absence of any commercial or financial relationships that could be construed as a potential conflict of interest.
